# Biliary Fascioliasis: A Scare During Endoscopic Retrograde Cholangiopancreatography

**DOI:** 10.14309/crj.0000000000000630

**Published:** 2021-07-21

**Authors:** Ricardo Valões, Nilton Maiolini Bonadeo, Pedro de Souza Quevedo, Anna Laura Duro Barp, Fernando Fornari

**Affiliations:** 1 Gatroenterology Department, Hospital São Vicente de Paulo, Passo Fundo, RS, Brazil; 2 Parasitology, Parasitic Diseases and Zoonoses, and Public Health, Universidade Federal do Sul e Sudeste do Pará, Nova Marabá, Marabá, PA, Brazil; 3 School of Medicine, University of Passo Fundo, Passo Fundo, RS, Brazil; 4 Gastroenterology Department, School of Medicine, University of Passo Fundo, Passo Fundo, RS, Brazil

## CASE REPORT

**Figure 1.**
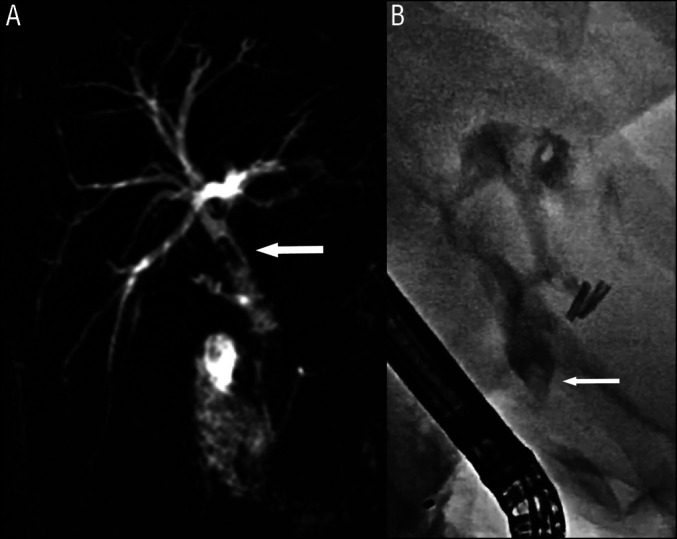
(A) Magnetic resonance cholangiopancreatography showing a hypointense filling defect (white arrow) into the distal part of the common bile duct, mimicking a gallstone. (B) Endoscopic retrograde cholangiopancreatography showing dilatation of the common bile duct, with radiolucent filling defects.

**Figure 2.**
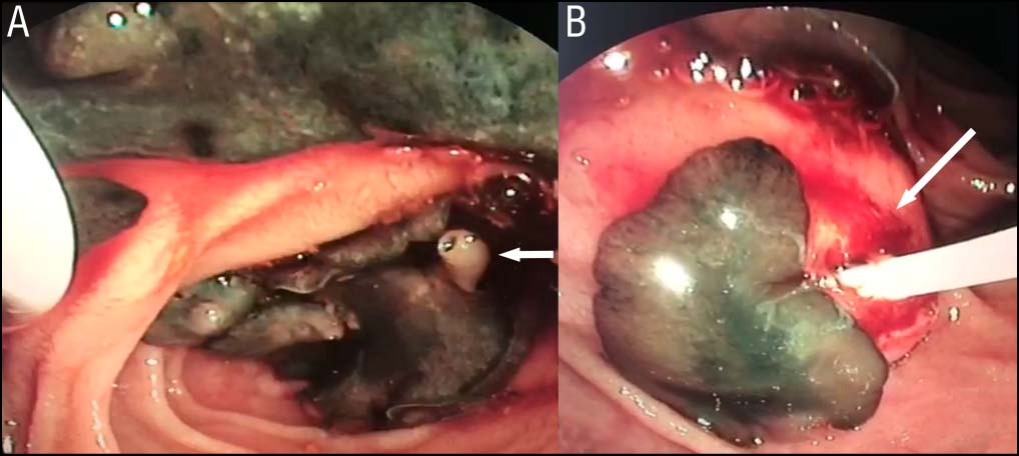
(A) Larvae of *Fasciola hepatica* in the duodenal lumen, with its suction cup indicated by the arrow. (B) The picture shows the balloon catheter (arrow) used to remove worms from the common bile duct.

A 44-year-old woman presents with icterus, abdominal pain, nausea, and fever. On examination, the vital signs were normal. The abdomen was soft, with moderate tenderness in the right upper quadrant. Blood tests showed a normal white cell count (6,290 per mm^3^) with eosinophilia (22%) and increased levels of total bilirubin (4.8 mg/dL), alkaline phosphatase (132 U/L), and alanine aminotransferase (101 U/L). The magnetic resonance cholangiopancreatography showed an enlarged common bile duct (1.1 cm), containing hypointense and heterogeneous material, suggestive of choledocholithiasis (Figure 1). The patient underwent an endoscopic retrograde cholangiopancreatography, which demonstrated radiolucent filling defects into the common bile duct (Figure 1). After sphincterotomy, the endoscopist observed the drainage of several live larvae from the biliary duct, similar to slugs (Figure 2). Fecal analysis confirmed the presence of eggs of *Fasciola* sp. On further history, the patient was discovered to be a nutritionist, reporting regular consumption of watercress, the likely source of fascioliasis. She was treated with triclabendazole for 2 days with prompt recovery.

The images illustrate a rare case of biliary fascioliasis, an infection caused by *Fasciola hepatica*, with worldwide distribution.^1^ It is transmitted to humans by watercress intake contaminated with sheep feces.^2^ The involvement of the biliary tract can occur in chronic *Fasciola hepatica* infection, manifested by cholestasis or mimicking cholangiocarcinoma.^1,3^ A high degree of suspicion is necessary for the diagnosis in the presence of abdominal pain, hepatomegaly, and eosinophilia. The identification of eggs in the stool confirms the infection.^3^ Triclabendazole is the best therapeutic option, with 10 mg/kg in a single dose for 2 days.^2^

## DISCLOSURES

Author contributions: All authors contributed equally to this manuscript. R. Valões is the article guarantor.

Financial disclosure: None to report.

Informed consent was obtained for this case report.
